# Seasonal and ontogenetic variation of skin microbial communities and relationships to natural disease dynamics in declining amphibians

**DOI:** 10.1098/rsos.140377

**Published:** 2015-07-15

**Authors:** Ana V. Longo, Anna E. Savage, Ian Hewson, Kelly R. Zamudio

**Affiliations:** 1 Department of Ecology and Evolutionary Biology, Cornell University, Ithaca, NY 14853, USA; 2 Department of Microbiology, Cornell University, Ithaca, NY 14853, USA; 3 Department of Biology, University of Central Florida, Orlando, FL 32816, USA

**Keywords:** community fingerprinting, 16S amplicon sequencing, dysbiosis, *Eleutherodactylus coqui*, *Lithobates yavapaiensis*, host–pathogen dynamics

## Abstract

Recently, microbiologists have focused on characterizing the probiotic role of skin bacteria for amphibians threatened by the fungal disease chytridiomycosis. However, the specific characteristics of microbial diversity required to maintain health or trigger disease are still not well understood in natural populations. We hypothesized that seasonal and developmental transitions affecting susceptibility to chytridiomycosis could also alter the stability of microbial assemblages. To test our hypothesis, we examined patterns of skin bacterial diversity in two species of declining amphibians (*Lithobates yavapaiensis* and *Eleutherodactylus coqui*) affected by the pathogenic fungus *Batrachochytrium dendrobatidis* (*Bd*). We focused on two important transitions that affect *Bd* susceptibility: ontogenetic (from juvenile to adult) shifts in *E. coqui* and seasonal (from summer to winter) shifts in *L. yavapaiensis*. We used a combination of community-fingerprinting analyses and 16S rRNA amplicon sequencing to quantify changes in bacterial diversity and assemblage composition between seasons and developmental stages, and to investigate the relationship between bacterial diversity and pathogen load. We found that winter-sampled frogs and juveniles, two states associated with increased *Bd* susceptibility, exhibited higher diversity compared with summer-sampled frogs and adult individuals. Our findings also revealed that hosts harbouring higher bacterial diversity carried lower *Bd* infections, providing support for the protective role of bacterial communities. Ongoing work to understand skin microbiome resilience after pathogen disturbance has the potential to identify key taxa involved in disease resistance.

## 1. Introduction

Microbial communities play an essential role in the maintenance of overall homoeostasis by modulating host immune responses, metabolism and physiological processes [1–3]. Therefore, any disturbance caused by, for example, the application of antibiotics, changes in diet, immune suppression or pathogen colonization can cause shifts in the relative abundance of resident microbial taxa, a condition known as dysbiosis [4]. Dysbiosis has been linked to chronic diseases in humans [5,6], but remains largely overlooked in the case of infectious diseases threatening wildlife [7]. Identifying conditions that disrupt stable host–microbe interactions is fundamental to understand how these associations evolved in natural environments to maintain health or to trigger disease.

For amphibians, the skin is a vital organ because it provides a protective barrier against pathogens, and also regulates physiological processes including osmoregulation, water absorption, respiration and thermoregulation [8]. Over the last few decades, chytridiomycosis, a skin disease caused by the fungal pathogen *Batrachochytrium dendrobatidis* (*Bd*, hereafter), has caused extinctions and severe declines in many amphibian populations across Australia, Europe and the Americas [9–12]. Some individuals have immunogenetic resistance and are able to clear *Bd* infection [13–15]. Others rely on abiotic and biotic factors to alleviate damage, such as increasing body temperature to reduce pathogen burden [16,17] or forming symbiotic associations with bacteria that indirectly provide resistance [18,19]. These factors are not mutually exclusive and may interact to determine disease outcome.

To date, studies characterizing the relationships between *Bd* and amphibian skin microbiota are limited to a few species of amphibians [20,21]. Thus, the functional role of microbial diversity for amphibians declining due to chytridiomycosis needs to be further explored. Amphibians often face periods of high *Bd* infection and mortality, especially during environmentally stressful times of the year or during early life stages [22,23]. These periods may alter the vigour of the host or the pathogen, and also the balance between ‘protective’ and ‘harmful’ skin bacteria leading to increased *Bd* infection rates. Because many bacteria isolated from amphibian skin express anti-*Bd* activity [24–28], dysbioses impeding the colonization, growth or reproduction of these protective microbes may predispose hosts to infection or promote higher rates of pathogen growth.

Here, we examine skin bacterial diversity in two very different amphibian species with well-characterized *Bd* infection dynamics: *Eleutherodactylus coqui* and *Lithobates yavapaiensis*. Although these species have contrasting life-history traits (direct versus aquatic development), geographical distributions (tropical versus temperate) and phylogenetic position (in two distantly related families, Eleutherodactylidae and Ranidae), both are susceptible to *Bd* infections [23,29], and continue to experience chytridiomycosis-associated mortalities [22,23]. In addition, these two species also show seasonal infection dynamics that consist of disease-mediated declines followed by limited population-level recovery [22,30–32]. By characterizing changes in microbial diversity across life-history stages or seasonal transitions, we can determine if periods of stress are associated with the occurrence of skin dysbioses, perhaps due to decreases in immune function [14,33]. A dysbiotic state may reflect a decrease in microbial diversity if some bacteria are favoured and dominate the community. Alternatively, a dysbiotic state may reveal an increase in microbial diversity driven by the colonization of transient bacteria. We predict the occurrence of dysbioses in amphibian hosts characterized by an increase in alpha and beta diversity during stressful times such as developmental changes and seasonal transitions.

To investigate associations between infection dynamics and skin bacterial diversity, we focused on two important transitions that affect *Bd* susceptibility: ontogenetic (from juvenile to adult) shifts in *E. coqui* and seasonal (from summer to winter) shifts in *L. yavapaiensis* [22,31]. Specifically, we expect higher microbial diversity values in juvenile *E. coqui* frogs, winter-sampled *L. yavapaiensis*, and in more highly *Bd-*infected frogs. Juvenile *E. coqui* frogs are almost three times more infected than adults [31,34], thus we predicted that the development of robust immune responses in adults would select for specific microbial taxa, thereby influencing community composition and structure. Similarly, *L. yavapaiensis* frogs carry significantly higher pathogen burdens and suffer mortality as a consequence of *Bd* infection during winter [22], thus we predicted that seasonal transitions would significantly influence community composition and structure.

We used community fingerprinting to quantify bacterial diversity and composition across species (*E. coqui* versus *L. yavapaiensis*), *Bd* infection status (positive versus negative), season (summer versus winter) and developmental stages (juvenile versus adult). We first compared inter- and intraspecific differences in microbial communities across these groups of frogs by focusing on three components of alpha diversity: richness, Shannon's diversity index and evenness. Second, we tested for changes in community structure by comparing ecological distances, which measured compositional differences in relative abundance and occurrence of bacterial constituents. Because community fingerprinting alone cannot distinguish the identity of microbial taxa and typically overestimates richness [35], we performed Illumina MiSeq 16S rRNA sequencing on a subset of *E. coqui* samples to investigate: (i) differences in core microbiome across host age, and (ii) the relationship of bacterial diversity and pathogen load. We focused our diversity estimates on the phylum Proteobacteria, which dominate skin microbial assemblages in amphibians [36–38] and comprise 80% of bacteria with known anti-*Bd* properties [28]. Our findings indicate that seasonal and ontogenetic transitions not only are important predictors of disease outcome, but also affect skin bacterial assemblages in wild amphibian populations.

## 2. Material and methods

### 2.1 DNA extraction and *Batrachochytrium dendrobatidis* quantification

We used swab samples to characterize skin microbial communities and determine the presence of *Bd* in multiple wild amphibian populations of *L. yavapaiensis* in Arizona (*N* individuals=37; electronic supplementary material, table S1) and *E. coqui* in Puerto Rico (*N* individuals=52; electronic supplementary material, table S1). We standardized the number of swab strokes per frog and body region and used a new pair of gloves to prevent sample cross-contamination [39]. Similar to studies of human skin microbiota [40], frogs were not rinsed before swabbing; thus, we assume that the microbial communities are a collection of resident and transient taxa. We preserved swabs in sterile vials with molecular grade ethanol (200 proof) upon collection in the field. Ethanol-based sample preservation can have mixed effects on prokaryotic DNA quality [41,42], but this is also true for other preservatives [43]; therefore, we compared only samples that were collected and preserved using identical methods. In the laboratory, we dried swabs in a vacuum centrifuge at room temperature and extracted DNA in 50 μl PrepMan Ultra extraction reagent (Life Technologies, Inc.). We followed standard methods for *Bd* DNA extraction and zoospore quantification [44], and performed amplifications in a ViiA7 qPCR machine using a *Bd* standard curve made from *Bd* isolate JEL427.

### 2.2 Microbial community fingerprinting

We used automated ribosomal intergenic spacer analysis (ARISA) to fingerprint skin bacterial communities from field-collected swabs [45]. We PCR-amplified the 16S-23S intergenic spacer region in the rRNA operon in bacteria using primers 1406F and 5^′^-fluorescently-labelled (TET) 23S-125R [45]. Due to the presence of inhibitors in the PrepMan DNA extracts, some samples required an additional cleaning and concentration step before amplification (Zymo Research). These cleaned samples were randomly distributed among samples and not associated with seasonal or age-related sampling events which might bias downstream analyses across groups. To quantify amplicon sizes, we ran equal amounts of PCR products for 5 h on an ABI 3730xl DNA Analyzer with a custom ROX-labelled standard [46]. We used the size, number and area of peaks in electropherograms to estimate the number and abundance of operational taxonomic units (OTUs) from each sample [45,47]. We analysed the GeneScan (Life Technologies, Inc.) output by discarding peaks less than five times the baseline as noise, and binning fingerprints using a shifting windows approach [47]. Two potential biases are present in ARISA: first, the length of the space region from unrelated microorganisms can be identical, and second, multiple operons within a single genome can differ in length [45]. Despite these shortcomings, community fingerprinting still remains a legitimate approach to understand general patterns of microbial diversity [48].

### 2.3 Statistical analyses using automated ribosomal intergenic spacer analysis

We evaluated skin microbial diversity using alpha diversity measures of richness, evenness and abundance (OTU richness, Shannon's diversity *H*^′^ and Pielou's evenness *J*^′^ [49]). We performed independent *t*-tests with pooled variances to test for mean differences in microbial diversity measures between species. In addition, we carried out separate multivariate analyses of variance (MANOVA) by species to test for potential effects of developmental stage (*E. coqui*) or season (*L. yavapaiensis*) and their interaction with *Bd* infection on microbial diversity measures [richness+*H*^′^+*J*^′^∼developmental stage (or season in *L. yavapaiensis*)×*Bd* infection (presence or absence)]. In previous studies, both variables were significantly associated with infection risk in these two species [22,31]. We implemented additional univariate *t*-tests with pooled variances to assess which diversity measures were driving the significant effects. All statistical analyses were carried out in R v. 3.0.2 [50].

We also evaluated potential shifts in skin microbial community structure driven by seasonal (summer to winter) or developmental (juvenile to adult) transitions. We first computed Bray–Curtis similarity indices among swab samples using function ‘vegdist’ in R package *vegan* [51]. Then, we used function ‘adonis’ to perform permutational multivariate analyses of variances (perMANOVA) testing the effects of developmental stage, season and *Bd* status on microbial community composition, using populations or species as strata. To confirm similar variance among groups, which is required to satisfy perMANOVA assumptions, we performed separate analyses of multivariate homogeneity of group dispersions (variances) by each variable using function ‘betadisper’. To visualize significant results, we used function ‘metaMDS’, which creates a non-metric multidimensional scaling (NMDS) plot. Finally, we illustrated the number of shared and unique OTUs by species, developmental stage and season using Venn diagrams.

### 2.4 16S V4 amplification and sequencing

To provide a taxonomic baseline for the OTUs identified by ARISA, we further analysed a subset of samples from each species (electronic supplementary material, table S1). Because the same DNA extracts were simultaneously used for *Bd* quantification and community fingerprinting, we did not have enough material to sequence every sample from the ARISA dataset. We performed paired-end 16S microbial community sequencing [52] on the Illumina MiSeq platform (2×250 bp) at the Genomics facility at Cornell University. We only used forward sequence reads for phylotype assignment. We PCR-amplified the V4 region of the 16S ribosomal RNA using universal bacterial/archaeal primers 515F/806R [53]. Briefly, triplicate PCR-amplifications and negative (no template) controls contained 10 μl 5-Prime Hot Master Mix (5-Prime Inc.), 13 μl water, 0.5 μl of 10 μM solution of each primer and 2 μl undiluted DNA template or water. Thermocycler conditions included the following steps: denaturation for 3 min at 94°C; followed by 35 cycles of 45 s at 94°C, 60 s at 50°C and 90 s at 72°C; and a final extension for 10 min at 72°C. PCR products were visualized on a 1.5% agarose gel and quantified using a Qubit^®^ double strand DNA high-sensitivity assay (Life Technologies, Inc.). We pooled 50 ng of all PCR products into a single vial and cleaned it using ChargeSwitch PCR clean-up kit (Invitrogen, Inc.) before sequencing. To ensure that our estimates of microbial diversity from field-collected swabs were not biased by *Bd* load, we blasted all the universal bacterial primer sets used in this study to the *Bd* genome and found no matches.

We analysed sequences and assigned phylotypes de novo using the quantitative insights into microbial ecology (QIIME) default pipeline v. 1.7.0 [54]. Briefly, sequences were filtered for quality, clustered into OTUs based on 97% sequence similarity with *uclust* using greengenes database (May 2013), aligned using PyNAST and used to infer a phylogenetic tree [54]. We filtered out phylotypes containing 0.001% of the total sequences [38], as well as samples with low coverage (less than 5000 sequences). We rarefied all samples to 5000 reads for alpha diversity analyses using 20 iterations.

We obtained a total of 1 179 178 16S rRNA tag sequence reads from 25 skin swab samples. This subset of samples achieved an average sequencing depth of 60 843 reads (min: 2823; max: 285 403) for adult *E. coqui* (*N*=15) versus 30 436 reads (min: 3048; max: 27 485) for juvenile frogs (*N*=6). Unfortunately, due to insufficient template material, we were only able to sequence four winter-collected *L. yavapaiensis* with an average of 20 976 reads (min: 7330; max: 43 217) per sample, precluding phylotype analyses between seasons. After applying the 0.001% abundance filter, we annotated 5206 unique phylotypes based on 97% sequence similarity with QIIME [54] from a total of 954 212 reads (electronic supplementary material, figure S1). We deposited sequences, phylotype table and mapping file in Dryad (http://dx.doi.org/10.5061/dryad.7v81b). Three individuals (one *L. yavapaiensis*, and an *E. coqui* adult and juvenile) were removed from analyses due to low coverage (more than 5000 reads).

### 2.5 Diversity analyses using 16S amplicon sequences

To avoid confusion with ARISA diversity results, we refer to all data generated by Illumina sequencing as phylotypes. To evaluate differences in microbial communities between species, we used QIIME to generate rarefied phylotype tables and compute alpha diversity metrics (i.e. phylotype richness, phylogenetic diversity, Shannon's diversity index, evenness and dominance). We rarefied tables at five 1000 read intervals only for interspecific comparisons. In addition, we focused on the phylum Proteobacteria (the most common phylum shared across amphibian species) to examine the distribution of phylotypes by taxonomic classes. Our objective was to determine if the distribution of Proteobacteria phylotypes was consistent between species, which may have contributed to differences in microbial diversity shown by community fingerprinting.

To evaluate which taxa were driving differences in microbial composition across developmental stages in *E. coqui*, we identified core phylotypes by considering taxa present in 95% of samples [55], which is more stringent than a previous study evaluating skin microbiomes in salamanders [56]. Given the low number of samples analysed, this 95% cut-off guaranteed that we were targeting common taxa consistently represented across adults and juveniles. In addition, we used *G*-tests to determine the relationship of particular core phylotype abundances with developmental stages. Finally, we investigated the relationship between Proteobacteria phylotype richness (number of distinct phylotypes), phylogenetic diversity, Shannon's diversity, dominance (1−Simpson index) and evenness with *Bd* load using Pearson correlations.

## 3. Results

### 3.1 Community fingerprinting of skin microbial diversity

**Figure 1.**
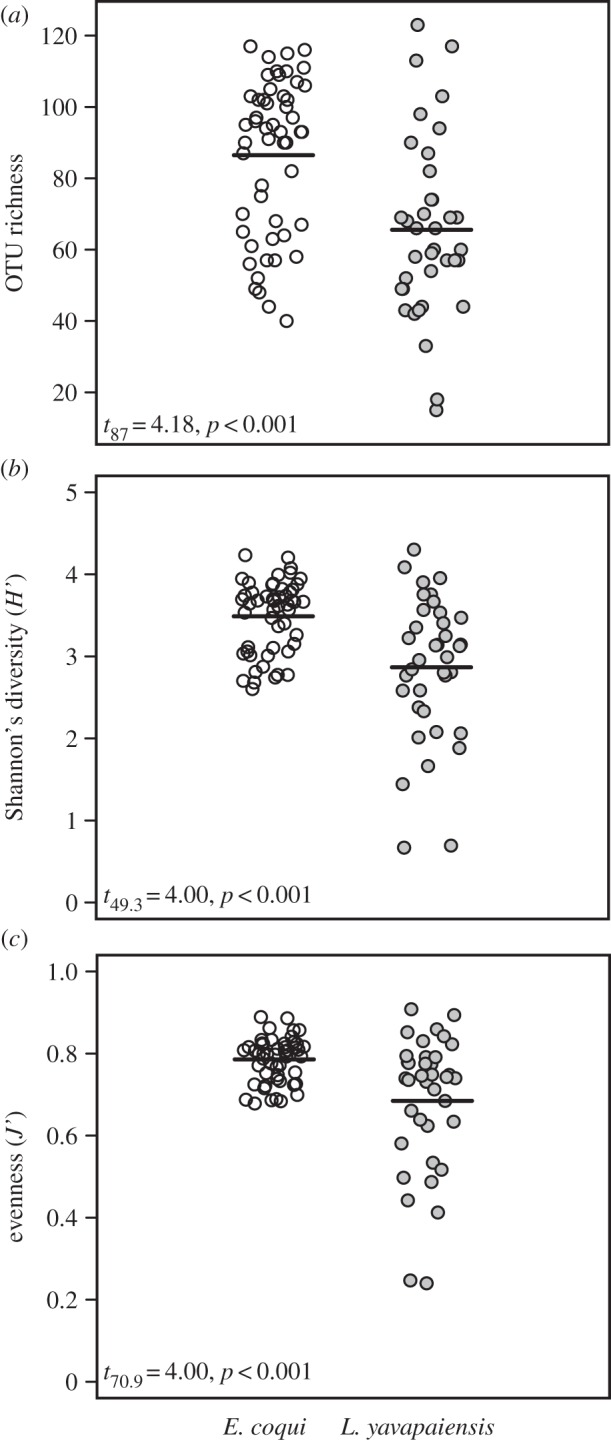
Mean interspecific differences in alpha diversity estimated by ARISA in *E. coqui* (open circles) and *L. yavapaiensis* (grey circles). (*a*) OTU richness, (*b*) Shannon's diversity and (*c*) Pielou's evenness.

Community fingerprinting (ARISA) revealed 180 distinct OTUs in our two focal species. The tropical frog *E. coqui* showed significantly higher alpha diversity measures (OTU richness, Shannon's diversity and evenness) than the temperate frog *L. yavapaiensis* (figure 1, all *p*<0.05). We found a significant effect of developmental stage (*Pillai's trace*=0.47, *p*<0.001) in *E. coqui* such that juveniles showed higher alpha diversity measures than adults, but no effects of *Bd* infection or their interactions (table 1). However, our power to detect an association with *Bd* in this species may have been limited by the very high frequency of positive frogs with low infections among our samples (90% prevalence: most frogs had infections of less than 100 *Bd* zoospores, table 1). By contrast, in *L. yavapaiensis*, MANOVAs detected a marginal, yet significant, effect of *Bd* infection (*Pillai's trace*=0.22, *p*=0.05). We found that *Bd*-infected *L. yavapaiensis* harboured a microbial community significantly more diverse and with more even relative abundances than *Bd*-uninfected frogs (table 1). In addition, the number of OTUs was significantly higher in frogs sampled during winter than during summer (table 1).

**Table 1.** Mean values of OTU richness, Shannon's diversity and evenness of microbial communities generated from ARISA fingerprinting of *E. coqui* and *L. yavapaiensis*skin swabs. Italicized values indicate significantly different means among groups, and asterisks denote *t*-test significance (*p<0.05, ^**^p<0.01 and ^***^p<0.001).

	*E. coqui*				*L. yavapaiensis*			
	infected	uninfected	juvenile	adult	infected	uninfected	winter	summer
	(*N*=47)	(*N*=5)	(*N*=21)	(*N*=31)	(*N*=20)	(*N*=17)	(*N*=15)	(*N*=22)
OTU richness	86.7	84.4	*103*.8^***^	*74*.*7*	72.8	57.1	*76*.1*	*58*.*4*
Shannon's diversity (*H*^′^)	3.5	3.37	*3*.83^***^	*3*.*25*	*3*.*19*	*2*.48^**^	3.18	2.66
evenness (*J*^′^)	0.79	0.76	*0*.83^***^	*0*.*76*	*0*.*75*	*0*.61^**^	0.73	0.65

**Figure 2.**
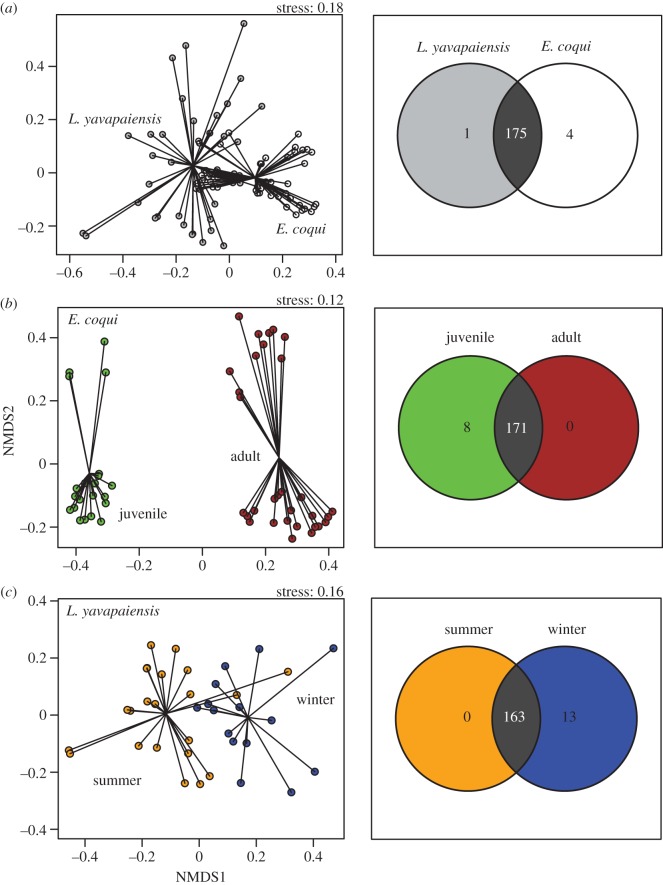
NMDS plots using Bray–Curtis distance matrices generated with ARISA show differences in community composition (beta diversity) by (*a*) species: *E. coqui* versus *L. yavapaiensis*, (*b*) life stage in *E. coqui*: juveniles versus adults and (*c*) season in *L. yavapaiensis*: summer versus winter. Venn diagrams show the number of shared and unique OTUs in each comparison.

Our analyses of skin microbial community composition (beta diversity) also showed significant differences among individuals differing in *Bd* status, species, developmental stage and season (figure 2). *Bd* infection was associated with changes in microbial community composition (*F*_1,88_=3.67, *p*<0.01, *r*^2^=0.04) but only explained 4% of the variation. Looking further at microbial communities by host species, we found that developmental stage explained most variation in microbial community composition (*F*_1,51_=24.7, *p*<0.001, *r*^2^=0.31) in *E. coqui*, followed by *Bd* infection status (*F*_1,51_=2.39, *p*<0.01, *r*^2^=0.03), and the interaction between developmental stage and *Bd* infection status (*F*_1,51_=3.39, *p*<0.001, *r*^2^=0.04). Similarly, for *L. yavapaiensis*, the season of capture (summer versus winter) significantly contributed to microbial community structure (*F*_1,36_=4.69, *p*<0.001, *r*^2^=0.12), but the influence of *Bd* infection was weaker (*F*_1,36_=1.51, *p*=0.07, *r*^2^=0.04), as well as the interaction between season and *Bd* infection status (*F*_1,36_=1.25, *p*=0.16, *r*^2^=0.03).

Most OTUs were shared between species; nonetheless, NMDS plots showed little overlap in microbial community structure (figure 2), with different beta dispersion between species (figure 2*a*). These patterns result from high variation in the relative abundance of particular taxa between species, which contributes to differences in evenness (figure 1). Partitioning samples by developmental stage in *E. coqui* revealed no overlap in community structure (figure 2*b*). In *L. yavapaiensis*, the seasonal difference in community structure was also evident in NMDS plots (figure 2*c*), as adult frogs gained 13 unique OTUs from summer to winter.

### 3.2 Identifying bacterial taxa driving diversity patterns

After rarefaction, we found a total of 754 unique phylotypes for *L. yavapaiensis* and 2729 for *E. coqui*, in addition to 1085 phylotypes that were shared between species (electronic supplementary material, figure S1). In contrast to ARISA diversity results, *L. yavapaiensis* skin harboured a greater microbial diversity relative to *E. coqui*, in phylotype richness, phylogenetic diversity, evenness and Shannon's diversity at all five rarefaction intervals (electronic supplementary material, figure S2). However, given that these findings are based on a limited number of samples (four individuals of *L. yavapaiensis*), the results from these analyses need to be interpreted with caution. Rarefaction curves of phylotype richness on both species did not reach saturation at 5000 sequences (electronic supplementary material, figure S2), suggesting that microbial diversity might be higher than reported in this study. By contrast, rarefaction curves for phylogenetic diversity reached a plateau, indicating that we captured most of the evolutionary divergence present in this dynamic ecosystem (electronic supplementary material, figure S2).

**Figure 3.**
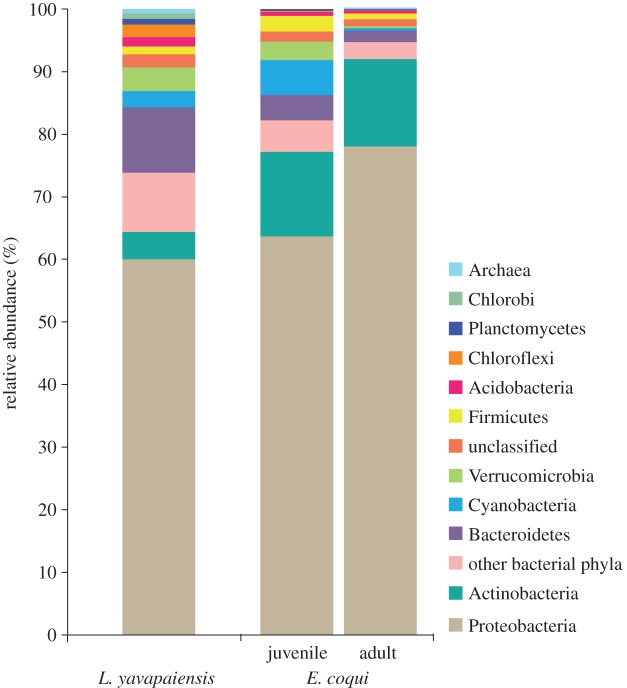
Relative abundance of 16S V4 amplicons by major phyla after rarefaction at 5000 reads per individual.

**Figure 4.**
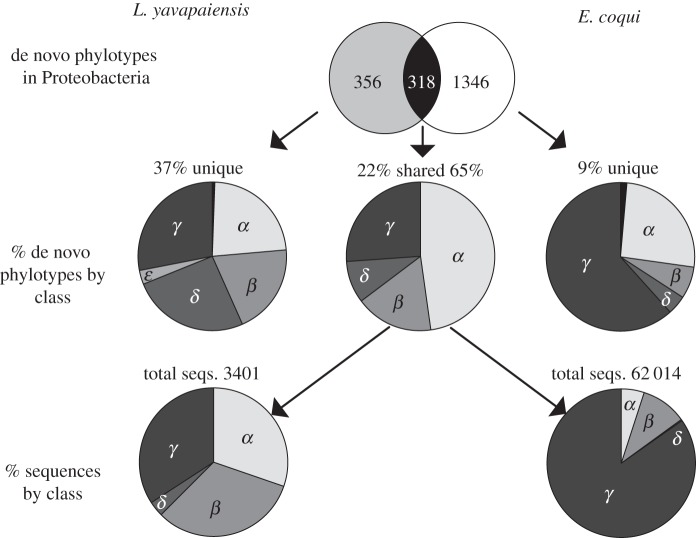
Relative abundance and distribution of 16S V4 Proteobacteria phylotypes by class after rarefaction at 5000 reads per individual. Venn diagram shows the total number of unique or shared phylotype sequences by species, whereas the pie charts represent the distribution of reads by class. We considered *% unique* as the total number of unique Proteobacteria reads for each species divided by total number of sequences (% unique _*Ly*_=5483/15 000; % unique _*Ec*_=8325/95 000). Similarly, we considered *% shared* as the total number of Proteobacteria reads shared between species divided by the total number of sequences (% shared _*Ly*_=3401/15 000; % shared _*Ec*_= 62 014/95 000).

The phylum Proteobacteria comprised 65% of all classified sequences (after rarefaction), dominating microbial communities for both species (figure 3). The top three phylotypes present in *E. coqui* skin microbial communities were Pseudomonadaceae (30%), *Stenotrophomonas* sp. (19%) and Comamonadaceae (8%) (table 2). By contrast, the top three phylotypes in *L. yavapaiensis* were *Arcobacter* sp. (7.6%), Pseudomonaceae (2.2%) and *Methylomonas* sp. (2.2%) (table 2). Differences in identity and relative abundance of dominant taxa within the phylum Proteobacteria alone could have contributed to microbial diversity estimates generated by ARISA (figure 4). We found that the distribution of Proteobacteria phylotypes at the class level was not consistent between species. Although the frequency of class Alphaproteobacteria was similar for unique phylotypes, Gammaproteobacteria phylotypes dominated in *E. coqui* (figure 4). For shared phylotypes, more sequences were classified as Gammaproteobacteria for both species (figure 4). However, in *E. coqui* Gammaproteobacteria were disproportionately represented (figure 4), whereas in *L. yavapaiensis* Alphaproteobacteria and Betaproteobacteria were represented in similar proportions.

**Table 2.** Mean relative abundance of the top five phylotypes for each species based on rarefaction at 5000 reads per individual. Warmer colours (reds) represent higher relative abundance and cooler colours (whites) represent lower relative abundances. Zero abundance indicates that the phylotype was not present in the sample after rarefaction.

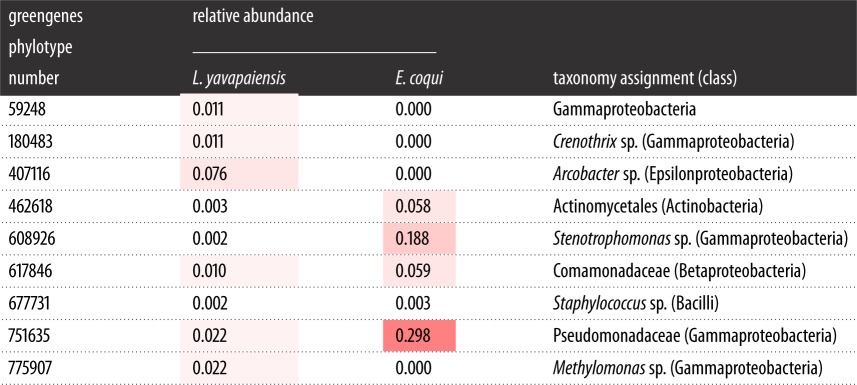

### 3.3 Characterizing the core microbiome in *Eleutherodactylus coqui*

We identified five core phylotypes in adult and juvenile *E. coqui* frogs (table 3). All phylotypes are part of the class Gammaproteobacteria. Interestingly, the two of these core phylotypes belong to the same family, Pseudomonadaceae, a pattern which is consistent with phylotypes isolated from red-backed salamanders [56]. A *G*-test revealed that all five core phylotypes had significantly different abundances across host developmental stage (table 3). Phylotypes assigned to *Acinetobacter johnsonii* and *Sphingomonas* sp. significantly dominated in juvenile samples (table 3), whereas Pseudomonadaceae, *Stenotrophomonas* sp. and *Erwinia* sp. had higher relative abundances in *E. coqui* adults.

**Table 3.** Mean relative abundance of the core phylotypes for *E. coqui* juveniles and adults based on rarefaction at 5000 reads per individual. Warmer colours (red) represent higher relative abundances and cooler colours (white) represent lower relative abundances.

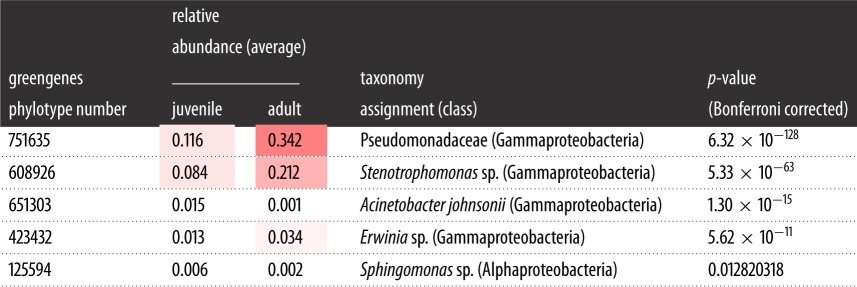

### 3.4 Linking Proteobacteria diversity to pathogen infection in *Eleutherodactylus coqui*

**Figure 5.**
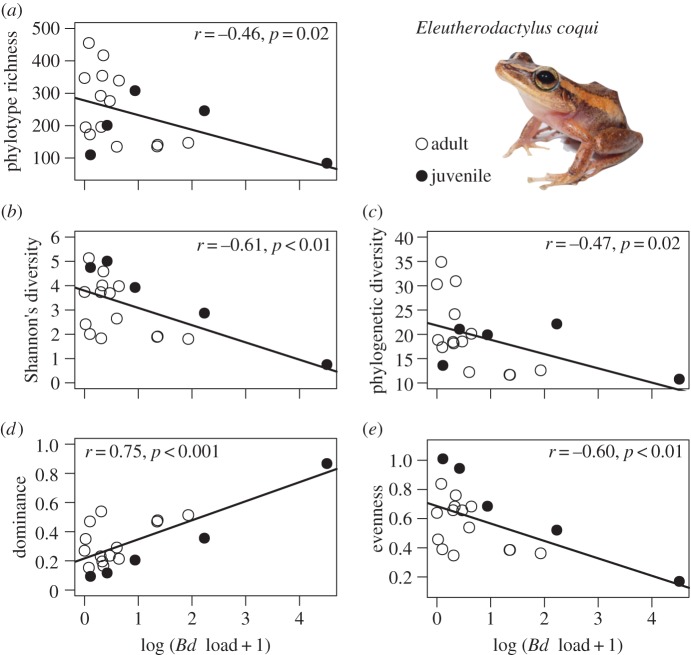
Correlations of *Bd* load (number of zoospore genomic equivalents) in *E. coqui* versus Proteobacteria (*a*) phylotype richness, (*b*) Shannon's diversity, (*c*) phylogenetic diversity, (*d*) dominance and (*e*) evenness. Photo of *E. coqui* by Alberto L. López-Torres.

Proteobacteria had a strong presence in skin microbial communities in both species; however, we were only able to analyse relationships with *Bd* infection for *E. coqui*. We detected significant negative correlations between the number of *Bd* zoospores on a host (infection load) and Proteobacteria phylotype richness, Shannon's diversity, phylogenetic diversity and evenness (figure 5*a*–*e*), even after removing one extreme outlier (log_10_ (*Bd* load+1)>4). By contrast, phylotype dominance (i.e. probability of randomly selecting two individuals from the same consensus lineage) positively correlated with *Bd* load (figure 5*d*). Analyses using the same samples from the ARISA dataset did not show these patterns (results not shown).

## 4. Discussion

Our results underscore that ontogenetic shifts for *E. coqui* and seasonal transitions for *L. yavapaiensis* are important factors determining skin bacterial diversity and community structure in two unrelated amphibian species affected by chytridiomycosis. In *E. coqui*, alpha diversity measures estimated by ARISA (OTU richness, diversity and evenness) were significantly higher in juveniles than in adults (table 1), concordant with their increased susceptibility to chytridiomycosis [31,34]. Similarly in *L. yavapaiensis*, adult skin swabs collected during winter months showed significantly higher OTU richness (table 1), corresponding to periods when frogs are most prone to *Bd* infection and associated mortality [22]. Assuming that ontogenetic and seasonal disease dynamics are at least in part driven by changes in immune responses, we expected to find a strong association of *Bd* infection on alpha and beta microbial diversity using both ARISA and sequencing. However, we found conflicting differences in host skin microbiota with *Bd* infection depending on the method used to assess microbial diversity. The first conflicting difference was found in interspecific comparisons, *L. yavapaiensis* showing higher diversity but only when using amplicon sequencing (electronic supplementary material, figure S2). The second difference was found in *E. coqui*, where we detected higher diversity values in juveniles, but only using ARISA (figure 5). Our study was not designed to directly compare results of ARISA and amplicon sequencing, because all samples were not available for both methods. Rather, we discuss how diversity patterns may arise under contrasting seasonal and ontogenetic states using these datasets independently.

### 4.1 Microbial shifts between seasons in *Lithobates yavapaiensis*

We found that *L. yavapaiensis* increased microbial richness from summer to winter. At this time, new bacterial taxa possibly colonized the skin as evidenced by the addition of 13 OTUs not present in summer (figure 2*c*). This could result from a number of temperature-dependent host–pathogen responses. First, temperate amphibians depend on specific temperature ranges to achieve optimal levels of immunity to fight infections [33]. In the case of *L. yavapaiensis*, we know that this species carries adaptive immune determinants associated with survival after *Bd* infection [13]. However, these immune factors may be ineffective in cool temperatures [57], leaving frogs unable to recognize and eliminate *Bd* and bacteria. Second, temperature directly influences *Bd* zoospore growth *in vitro* [58], but could also affect other skin microbes [59]. *Lithobates yavapaeiensis* lives in microhabitats where water temperatures exceed *Bd*'s upper temperature limit [60], which provides an additional defence strategy for the host. In the same way, temperature could alter microbial community dynamics in the skin or in the microbial source pool by halting growth, or enhancing colonization and/or displacement by certain bacterial species. For example, using 16S rRNA amplicon sequencing, we found that *Arcobacter* sp. was the most abundant phylotype in *L. yavapaiensis* (table 2). Members of genus *Arcobacter* are emergent pathogens and potential zoonotic agents [61], suggesting increased host susceptibility. Therefore, we should expect variability in putatively pathogenic constituents of microbial assemblages as a consequence of natural environmental fluctuations that impact both host physiology and immunity. Whether or not enhanced microbial colonization during winter evolved as an innate immune response to fight stronger pathogens such as *Bd* remains to be explored under other seasonal contexts (e.g. spring and autumn) and also using temperature-controlled trials.

Our hypotheses of increased microbial diversity were based on well-documented patterns of susceptibility to *Bd* for this species, in which chytridiomycosis is more prevalent in adults during winter [22]. Given that our comparisons were limited to adult *L. yavapaiensis*, we can be certain that increased microbial diversity during winter was not an ontogenetic-related sampling bias (i.e. sampling more juveniles during summer). However, we know that few juvenile *L. yavapaiensis* are active during winter [22], indicating that a shift in the demographic structure of the population could have indirect effects on microbial species turnover. This overlap of developmental classes in summer should increase richness and phylogenetic diversity of microbes, but our results showed the opposite pattern. Future studies will be essential to identify environmental sources of bacteria, and also to test for the effects of developmental stages across bacterial taxa in this species.

### 4.2 Microbial shifts in the absence of metamorphosis in *Eleutherodactylus coqui*

In organisms with biphasic life histories, immunological re-arrangements are most extreme during metamorphosis [62,63]. The ability to synthesize antibodies and antimicrobial peptides is reduced during development [62], which possibly alters host-associated microbial communities in the gut and skin [37,64]. Our focal species for the ontogenetic analysis, *E. coqui*, is a direct-developing frog that lacks a free-living tadpole phase and does not develop through metamorphosis in a strict sense [65]. Nonetheless, in our study host developmental stage accounted for 31% of the variation in skin microbial community composition (figure 2*b*), corroborating recent findings for amphibians with tadpole stages [37,64]. Although both developmental stages shared almost all OTUs identified by ARISA, community structure and diversity were significantly different between juveniles and adults (table 1 and figure 2*b*), caused by the loss of eight unique OTUs from juveniles and by changes in the relative abundance of bacterial taxa. Yet, we failed to detect these patterns using phylotype alpha diversity estimated from amplicon sequencing, perhaps due to smaller sample sizes or the level of taxonomic resolution possible with our data.

Given that the two methods showed distinct results, we cannot confirm that high bacterial diversity results from underdeveloped immune responses in young individuals in this direct-developing species, but our findings suggest some possible mechanisms for developmental shifts in skin-associated microbes. Studies in humans suggest that the role of skin microbes is to educate the adaptive immune system [66]; therefore, having increased alpha diversity at early ages might actually benefit the host in later life stages. In the gut, *Lithobates pipiens* tadpoles harbour a microbial community with higher phylogenetic diversity than adults [64], which also supports this hypothesis. However, taken together, these findings contrast with the patterns of increased phylogenetic diversity of skin microbes with developmental stage found for *Rana cascadae* [37]. Different selective events may be acting in the skin compared with the gut, because post-metamorphic individuals tend to use more terrestrial habitats and these contain higher microbial diversity than aquatic habitats [37].

### 4.3 Is skin microbial diversity related to disease outcome?

Skin microbial communities provide a resistance barrier to impede colonization of exogenous microbes by producing antimicrobial metabolites [67] or by inhibiting colonization sites. Because our samples are from field-collected individuals, we cannot distinguish if high microbial diversity in juveniles and winter-collected frogs is (i) part of an adaptive strategy for host survival during periods of stress, (ii) a consequence of lowered immunological responses, or (iii) results from differences in host behaviour or microbe physiology. In addition, these responses may not be mutually exclusive. Hosts establishing symbiotic associations often also exhibit reduced immune responses [68,69], which facilitates microbial colonization and persistence of beneficial bacteria. At the same time, opportunistic microbes can invade skin, possibly facilitated by temperature shifts, also resulting in high microbial diversity.

Our results highlight critical differences in fungal pathogen (*Bd*) associations with alpha and beta diversity of skin microbial communities. *Bd* infection status, considered here as a binary category, was not correlated with alpha diversity values estimated by ARISA in *E. coqui* (table 1). When we evaluated community composition and structure (beta diversity), the presence of *Bd*, although significant, explained much less of the variation than host developmental stage. Likewise, in *L. yavapaiensis*, *Bd*infection status did not influence richness, but was significantly correlated to the relative abundances of microbes, as measured by Shannon's diversity and Pielou's evenness (table 1). Therefore, *Bd* infection status had only a marginal effect on microbial community structure, possibly because both species are getting infected, clearing the pathogen, and becoming re-infected throughout the year [22,32].

By contrast, *Bd* infection intensity (a continuous variable measured as the number of zoospores in each sample) was more informative in correlations with alpha phylotype diversity (figure 5) in *E. coqui.* The phylum Proteobacteria dominated skin microbial communities and their phylotype richness and diversity were negatively correlated with *Bd* load (figure 5*a*–*e*), while phylotype dominance was positively correlated with pathogen load (figure 5*d*). When *Bd*colonizes amphibian skin, it disturbs skin stability, impedes electrolyte transport [70] and changes the environment for microbial communities [20], possibly leading to the observed reduction in phylotype richness and diversity (figure 5). Alternatively, higher phylotype diversity could saturate the skin, therefore limiting *Bd* growth or establishment, or increase the probability of anti-*Bd* bacteria as part of the core microbial communities. Our data do not allow us to distinguish between these two possibilities, but we do provide some evidence that Proteobacteria taxon dominance may be protective (figure 5*d*). The two most abundant phylotypes found on *E. coqui* belong to Proteobacteria and more specifically to the family Pseudomonadaceae, a family in which a number of members show antimicrobial activity [24,26,28]. These core taxa were consistently present in 95% of samples, yet varied in relative abundance between juveniles and adults (table 3). Therefore, it is possible that Pseudomonadaceae members contribute to differences in *Bd* susceptibility among hosts. The correlation between phylotype dominance and pathogen load should be investigated with controlled experiments designed specifically to evaluate microbial recruitment through time after *Bd* infection, including the quantification of total bacteria via qPCR. These experiments will identify whether or not *Bd* load is correlated with particular bacteria due their protective role against *Bd* or due to proliferation of specific microbial taxa during secondary infections.

## 5. Conclusion

We have demonstrated that developmental stage and seasonal changes are important factors influencing skin microbial communities in species that persist with enzootic infections of the amphibian-killing fungus. The influence of these two factors was greater than the presence or absence of *Bd*, indicating that environmental variables, and secondarily host immunity, play important roles in shaping microbial occurrence on the skin. We showed that increases in skin microbial diversity occurred during specific periods when hosts were most likely to be immune-suppressed. Higher microbial diversity can result from increased susceptibility due to infection, or as a mechanism to fight infections if hosts are actively recruiting beneficial microbes. Quantifying surface microbiomes and host immune function from eggs to adult, in the presence and absence of *Bd* infection, will allow us to fully characterize how the ontogeny of immune systems affects microbial colonization and persistence. At the same time, future research should quantify the temporal dynamics of microbial communities in the skin and the environment, as a step towards elucidating the role of disease versus microbial reservoirs as the underlying causes of shifts in community structure.

## Supplementary Material

Figure S1

## Supplementary Material

Figure S2

## Supplementary Material

Table S1

## Data accessibility

Mapping file, sequences, OTU table and community phylogenetic tree have been accessioned at the Dryad Digital Repository (http://dx.doi.org/10.5061/dryad.7v81b).
