# Supplementary material for: Seasonal and ontogenetic variation of skin microbial communities and relationships to natural disease dynamics in declining amphibians
Source: R Soc Open Sci. 2015 Jul 15;2(7):140377. doi: 10.1098/rsos.140377 (PMC4632566; doi:10.1098/rsos.140377)
Supplement: Figure S1 [file rsos140377supp1.pdf]

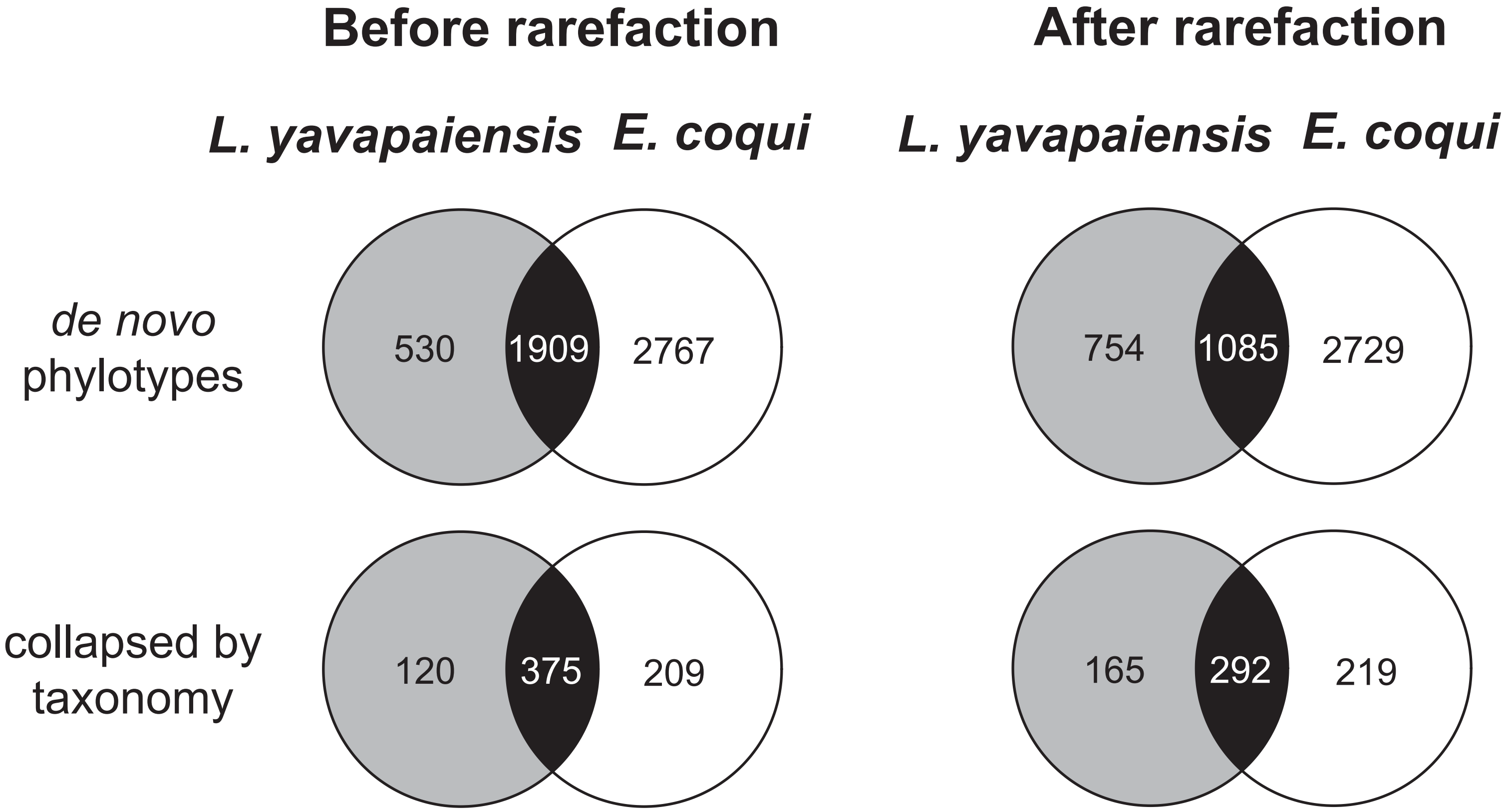

Before rarefaction
After rarefaction
L. yavapaiensis
E. coqui
L. yavapaiensis
E. coqui
de novo phylotypes
530
1909
2767
754
1085
2729
collapsed by taxonomy
120
375
209
165
292
219
