# Supplementary figures and images for: Seasonal and ontogenetic variation of skin microbial communities and relationships to natural disease dynamics in declining amphibians
Source: R Soc Open Sci. 2015 Jul 15;2(7):140377. doi: 10.1098/rsos.140377 (PMC4632566; doi:10.1098/rsos.140377)

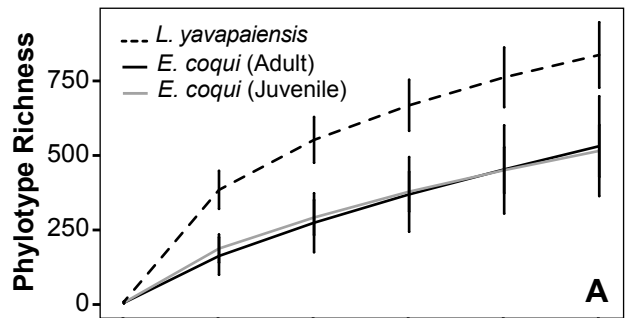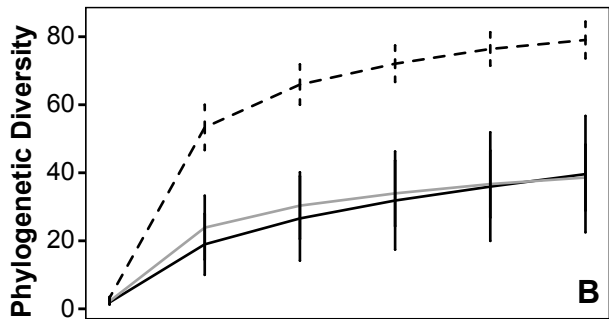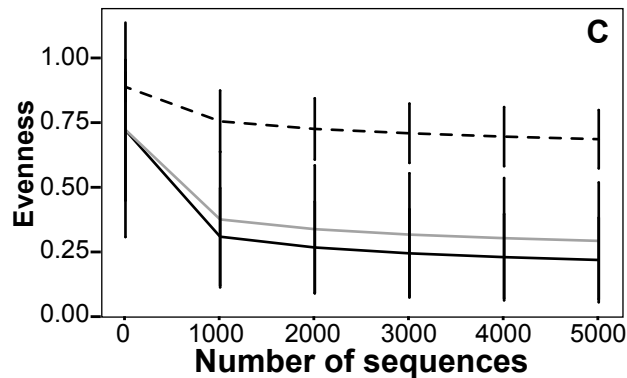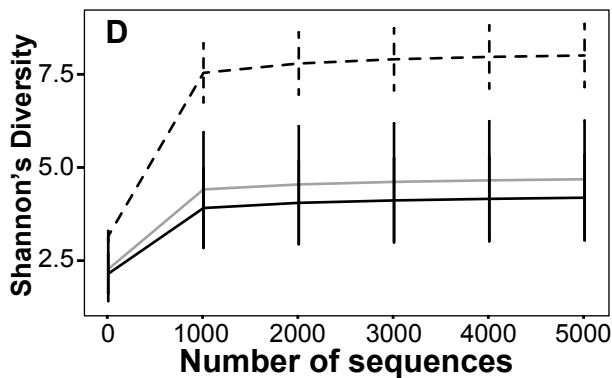

Supplement: Figure S2 [file rsos140377supp2.pdf]
