# Supplementary material for: Seasonal and ontogenetic variation of skin microbial communities and relationships to natural disease dynamics in declining amphibians
Source: R Soc Open Sci. 2015 Jul 15;2(7):140377. doi: 10.1098/rsos.140377 (PMC4632566; doi:10.1098/rsos.140377)
Supplement: Table S1 [file rsos140377supp3.docx]

| ***Eleutherodactylus coqui*** |  | **ARISA** | | **Illumina** | |
| --- | --- | --- | --- | --- | --- |
| **Population** | **Coordinates** | **Adult** | **Juvenile** | **Adult** | **Juvenile** |
| El Yunque | 18.299°N, 65.779°W | 13 | 11 | 7 | 4 |
| Carite | 18.093°N, 66.031°W | 2 | 2 | 1 | 1 |
| Toro Negro | 18.172°N, 66.489°W | 10 | 5 | 6 | 1 |
| Maricao | 18.148°N, 66.988°W | 6 | 3 | 1 | - |
| **Total** |  | **31** | **21** | **15** | **6** |
|  |  |  |  |  |  |
|  |  |  |  |  |  |
| ***Lithobates yavapaiensis*** |  |  |  |  |  |
| **Population** | **Coordinates** | **Summer** | **Winter** | **Summer** | **Winter** |
| Aravaipa Canyon | 32.877°N, 110.392°W | 7 | 7 | - | - |
| Aliso Spring | 31.582°N, 111.100°W | 4 | 1 | - | 3 |
| Cienega Creek | 31.998°N, 110.596°W | 3 | 7 | - | 1 |
| Muleshoe Ranch | 32.337°N, 110.239°W | 8 | - | - | - |
| **Total** |  | **22** | **15** | **0** | **4** |

**Table S1.** Number of individuals by population analyzed using ARISA microbial community fingerprinting (ARISA) and Illumina 16S V4 amplicon sequencing. All *Eleutherodactylus coqui* samples were collected in January 2011. *Lithobates yavapaiensis* samples were collected in the summer 2008 and winter 2009.
